# Supplementary material for: The causal relationship between the human gut microbiota and pyogenic arthritis: a Mendelian randomization study
Source: Front Cell Infect Microbiol. 2024 Nov 26;14:1452480. doi: 10.3389/fcimb.2024.1452480 (PMC11629706; doi:10.3389/fcimb.2024.1452480)
Supplement: Supplementary file 1 [file DataSheet1.zip › mendelian test/ebi-a-GCST90027739.csv_leave_one_out.pdf]

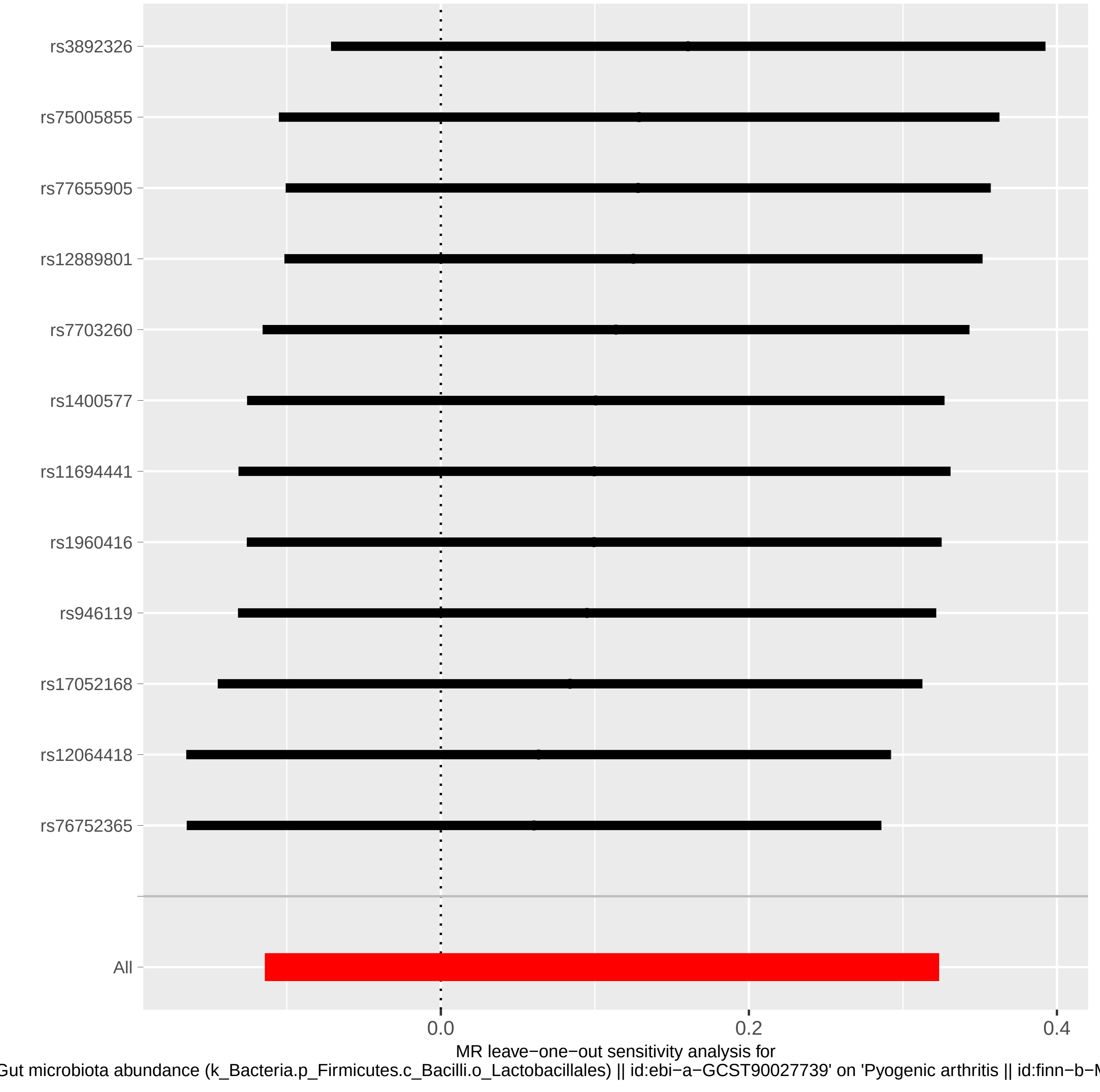
rs3892326
rs75005855
rs77655905
rs12889801
rs7703260
rs1400577
rs11694441
rs1960416
rs946119
rs17052168
rs12064418
rs76752365
All
0.0
0.2
0.4
MR leave−one−out sensitivity analysis for
Gut microbiota abundance (k_Bacteria.p_Firmicutes.c_Bacilli.o_Lactobacillales) || id:ebi−a−GCST90027739' on 'Pyogenic arthritis || id:finn−b−M
